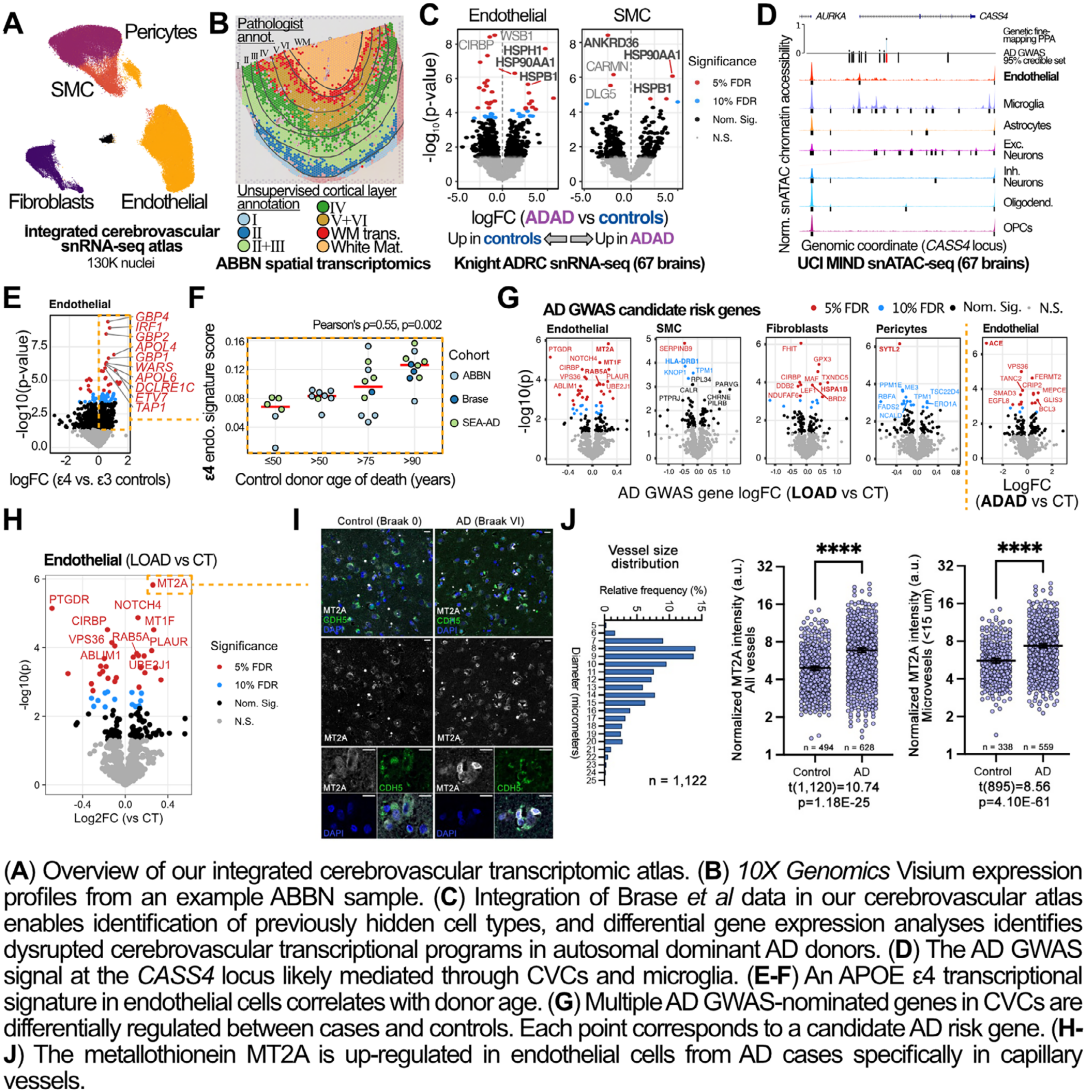# Dissecting the complex interplay between AD and cerebrovascular dysfunction in human brains across the genetic and molecular layers through functional genomics

**DOI:** 10.1002/alz70855_101122

**Published:** 2025-12-23

**Authors:** Ricardo D'Oliveira, Taylor Bertucci, Elanur Yilmaz, Ekaterina Aladyeva, Bruno A. Benitez, Caghan Kizil, Greg T Sutherland, Celeste M. Karch, Sally Temple, Oscar Harari

**Affiliations:** ^1^ Washington University School of Medicine, St. Louis, MO, USA; ^2^ Neural Stem Cell Institute, Albany, NY, USA; ^3^ Columbia University Irving Medical Center, New York, NY, USA; ^4^ Ohio State University Wexner Medical Center, Columbus, OH, USA; ^5^ Beth Israel Deaconess Medical Center, Boston, MA, USA; ^6^ University of Sydney, Camperdown, NSW, Australia

## Abstract

**Background:**

Cerebrovascular disorders are associated with an increased risk for neurodegenerative diseases, including Alzheimer's disease (AD). However, the mechanisms by which cerebrovascular dysfunction contributes to neurodegeneration are poorly understood. We aim to dissect the complex interplay between vascular dysfunction and AD to molecular features in cerebrovascular cells (CVCs) through deep multi‐omic profiling of human brains.

**Method:**

We have previously performed single‐nucleus transcriptomic profiles (snRNA‐seq) and chromatin accessibility (snATAC‐seq) of parietal cortex from healthy and AD donors from the Australian Brain Bank Network (ABBN; *n* = 72 brains), which are richly annotated for cerebrovascular phenotypes, including cerebral amyloid angiopathy (CAA). In parallel, we generated spatially resolved transcriptomic profiles for a subset of these samples. We integrated this data with seven public snRNA‐seq datasets to create a comprehensive multi‐omic cerebrovascular atlas.

**Result:**

Our cerebrovascular atlas encompassed >133K nuclei across major CVC types. This high resolution identified perturbed CVC transcriptional programs between cases and controls, including changes specific to rare AD risk variants. We identified perturbed regulatory programs associated with CAA in large‐diameter endothelial cells and capillary‐specific changes related to late‐onset AD, including up‐regulation of metallothionein genes. We validated these findings in human brains using immunohistochemistry. In addition, we identified an age‐associated CVC transcriptional signature linked to APOE4 detectable before disease onset, suggesting that APOE4 associates with accelerated vascular aging. Lastly, we used snATAC‐seq to prioritize multiple independent AD risk loci, including APP and APOE, where at least one fine‐mapped risk variant (95% credible set) overlapped a regulatory element active in vascular cells. Finally, we observed extensive transcriptional changes in putative AD‐risk genes in all CVCs subtypes, supporting a shared genetic risk between AD and cerebrovascular disease but suggesting the shared risk is mediated in a cell type‐specific manner.

**Conclusion:**

Our work provides novel insights into the links between cerebrovascular dysfunction and AD and identifies genes and regulatory elements mediating AD genetic risk through CVCs.